# Divergence and adaptive evolution of the gibberellin oxidase genes in plants

**DOI:** 10.1186/s12862-015-0490-2

**Published:** 2015-09-29

**Authors:** Yuan Huang, Xi Wang, Song Ge, Guang-Yuan Rao

**Affiliations:** College of Life Sciences, Peking University, Beijing, 100871 China; State Key Laboratory of Systematic and Evolutionary Botany, Institute of Botany, Chinese Academy of Sciences, Beijing, 100093 China

**Keywords:** Characteristic motif, Functional divergence, Gibberellin, Phytohormones, Positive selection, 2-oxoglutarate-dependent dioxygenase

## Abstract

**Background:**

The important phytohormone gibberellins (GAs) play key roles in various developmental processes. GA oxidases (GAoxs) are critical enzymes in GA synthesis pathway, but their classification, evolutionary history and the forces driving the evolution of plant GAox genes remain poorly understood.

**Results:**

This study provides the first large-scale evolutionary analysis of GAox genes in plants by using an extensive whole-genome dataset of 41 species, representing green algae, bryophytes, pteridophyte, and seed plants. We defined eight subfamilies under the GAox family, namely C_19_-GA2ox, C_20_-GA2ox, GA20ox,GA3ox, GAox-A, GAox-B, GAox-C and GAox-D. Of these, subfamilies GAox-A, GAox-B, GAox-C and GAox-D are described for the first time. On the basis of phylogenetic analyses and characteristic motifs of GAox genes, we demonstrated a rapid expansion and functional divergence of the GAox genes during the diversification of land plants. We also detected the subfamily-specific motifs and potential sites of some GAox genes, which might have evolved under positive selection.

**Conclusions:**

GAox genes originated very early—before the divergence of bryophytes and the vascular plants and the diversification of GAox genes is associated with the functional divergence and could be driven by positive selection. Our study not only provides information on the classification of GAox genes, but also facilitates the further functional characterization and analysis of GA oxidases.

**Electronic supplementary material:**

The online version of this article (doi:10.1186/s12862-015-0490-2) contains supplementary material, which is available to authorized users.

## Background

The gibberellins (GAs), one of important phytohormones, form a large family of diterpene hormones that are involved in various growth and developmental processes in plants [1–6]. Among hundreds of plant GAs, only a few, such as GA1 and GA4 are bioactive [6]. The function of bioactive GAs generally depends on their concentration in a given tissue, and this is mainly affected by their biosynthesis and/or deactivation [7]. In Arabidopsis, the synthesis pathway of GAs has been elucidated, and most genes encoding enzymes to catalyze GA biosynthesis have been identified [7–11]. In the GA synthesis pathway, three classes of enzyme (i.e., Terpene synthases (TPSs), P450s and GA oxidases (GAoxs)) are required for the biosynthesis of bioactive GAs from geranylgeranyl diphosphate (GGDP, a common precursor of terpenes in plants), and the pathway can be divided into two main steps: early and late. The early steps are catalyzed by a series of enzymes, i.e., CPS, KS, KO, and KAO, which are all encoded by single genes. The enzymes catalyzing later steps (i.e., GA2 oxidase, GA20 oxidase, and GA3 oxidase) are encoded by small gene families, and these oxidases are all soluble 2-oxoglutarate-dependent dioxygenases (2OGDs) [7, 10, 12, 13]. In comparison with the biosynthesis enzymes in the early steps, those in the later steps are differentially regulated by developmental and environmental cues, and these enzymes play key roles in the regulation of bioactive GA levels. For example, the loss-of-function in GA20 oxidase (GA20ox) and GA3 oxidase (GA3ox) can generate dwarf phenotypes, such as the well-known Green Revolution *sd*-*1* [10, 14–17]. Interestingly, the function of GA2 oxidase (GA2ox) is to decrease the levels of active GAs rather than increasing the GA level as do GA20ox and GA3ox [18]. So far, most studies have focused on the functions of GA2ox, GA20ox and GA3ox [10, 12, 18–21]. However, little is known about the evolutionary history of the GAox genes [13, 22–24].

GAoxs are ubiquitous in vascular plants, but no GAs has ever been isolated and chemically identified from bryophytes [25–28]. Those authors hypothesized that GAs may have first appeared in ancient pteridophytes and that the hormonal signaling pathway developed later during the evolution of land plants. However, several putative GA biosynthetic genes have been found in the *Physcomitrella patens* genome, suggesting the GAs could appear in the earlier lineages of land plants [29, 30]. Although the pathways leading to production of bioactive GAs have been studied in plants [31–33], the evolutionary history of the GA synthetic pathway and its catalyzing enzyme genes, especially of GAox genes remains unclear.

The GAox family has been evolutionarily analyzed, but only focusing on the GA20ox, GA3ox and GA2ox subfamilies of a few angiosperm species [13, 23, 24]. Han and Zhu (2011) identified 61 GA oxidase genes from rice (*Oryza sativa*), Arabidopsis and soybean (*Glycine max*), and performed the phylogenetic analysis of these GAox genes. They were grouped into four clades (GA20ox, GA3ox, C_19_-GA2ox, and C_20_-GA2ox.), but their relationship remains unclear, especially of new putative GA20ox homologs. For example, four new genes were described under the GA20ox subfamily (i.e., *OsGA20ox5*, *OsGA20ox6*, *OsGA20ox7* and *OsGA20ox8*), but three of them (*OsGA20ox5*, *OsGA20ox6*, and *OsGA20ox8*) did not reside within the GA20ox subgroup according to their phylogenetic tree. Meanwhile, Han and Zhu (2011) showed that the GA2ox subfamily is not monophyletic [23], which was supported by Giacomelli and her collegues’ study [24]. In addition, there is controversy over the relationship between GA20ox, GA3ox, C_19_-GA2ox and C_20_-GA2ox [23, 24]. Giacomelli et al. (2013) found the sister relationship between C_19_-GA2ox and GA3ox, while the close relationship was found between GA3ox and C_20_-GA2ox in Han and Zhu’s study. Not only the relationship between GAox genes but the evolutionary history and expansion mechanisms of the GAox genes are also poorly understood.

The GAox family belongs to the 2OGDs superfamily. The catalytic core of GAoxs has a double-stranded β-helix (DSBH) fold containing a HX[DE] dyad (where X could be any amino acid) and a conserved carboxy-terminal histidine which together chelate a single iron atom. Specifically, the DSBH region has seven conserved strands that are common to all these kinds of proteins and is arranged in two sheets in a jelly-roll topology [34]. To date, the function of several GAox genes has been determined but their gene structure not studied systematically [10, 20, 35–40]. Of GA oxidases, GA 20ox was first isolated from cotyledons of immature pumpkin seeds by Lange et al. (1994) [41]. Structurally this gene has characteristic motifs that are highly conserved in 2OGDs. Later on, other GAox genes were isolated and characterized in plants, such as Carrizo citrange [42], poplar [43], *Phaseolus coccineus* and Arabidopsis [19], rice [38, 44, 45], spinach [37, 46], watermelon [39], pea [47, 48]. On the characteristic motifs of different GA oxidases, no consensus has come out. Meanwhile, neither diagnostic sequences nor gene structures have been established for identification of GAox subfamilies. For example, four GAox homologs were found in the *Physcomitrella patens* genome, but it is hard to conclude what they are and which gene subfamily they belong to [30]. Thus, characterizing the motif specific to every gene subfamily would be of particular value. In brief, the study of gene structures and conserved motifs will not only provide an insight into the classification of GAox genes, but also their evolutionary history and functional diversification.

Almost all aspects of the functional divergence of genes are in some way linked to gene duplications, which occur ubiquitously [49–54]. What was the evolutionary force driving the functional divergence of genes or gene families? Generally, gene duplications are thought to be an important precursor to the functional divergence of genes, and selection is the main evolutionary force driving gene function diversification [22, 52, 55–59]. We investigated whether the functional divergence of GAox genes was driven by positive selection, in particular for four newly described ones. We first analyzed the distribution of positively selected amino acids in the characteristic motifs of genes. Then, we used the programs FunDi and GroupSim to validate the hypothesis that the conserved domains were associated with divergence (change) in function among GAs, and performed the GroupSim to identify the specificity determining positions (SDPs) [60–62].

With the availability of more whole genome sequence data and new genomics tools developed in model plants, the study of GA biosynthetic pathways, GAox genes and their evolution is now feasible. To reach the above objectives, we performed a comprehensive analysis of the GAox gene homologs in 41 species, using whole genome sequencing data, covering the main lineages of green plants from green algae, bryophytes, pteridophytes and gymnosperms to angiosperms. First, a genome-wide search was conducted and GAox homologs identified. Then, we performed phylogenetic analysis to explore the evolutionary history of GAox genes and established the diagnostic features of conserved motifs for every GAox subfamily. Next, the expansion mechanism and evolutionary forces associated with the GAox genes were investigated by analyzing the evolutionary history, structure and functional divergence of these genes. Finally, we compared the GA biosynthesis pathways in bryophytes, pteridophytes and seed plants, to explore the evolution of pathways and their relationships with the adaptation of plants to terrestrial environments.

## Methods

### Sequence acquisition and compilation

In order to investigate the evolutionary history of the GAox family in plants, all possible sequences of GAox homologs need to be sourced from whole genome sequencing data. GAox belongs to the 2OGDs superfamily, which has a very conserved and characteristic 2OGDs motif (Pfam id, PF03171) [7, 34, 63]. Functional GAox genes of Arabidopsis and rice, and four newly found GAox genes (*OsGA20ox5*, *OsGA20ox6*, *OsGA20ox7*, and *OsGA20ox8*; Han and Zhu, 2011) were used as queries to BLAST against the whole genome sequences of 41 species. The sequences with the active site core of 2OGD, and the motifs such as NYYPXCXXP, LPWKET, LSWSEA were recognized as putative GAox genes. GAox homologs of each species were downloaded from the following databases: http://www.phytozome.net; http://congenie. org; http://www.Arabidopsis.org/; http://rice.plantbi-ology.msu.edu/; http://www.amborella.org/. Then, an extensive screening of databases (dbEST, GenBank) was performed using different BLAST algorithms to obtain all GAox homologs [64]. Finally, we manually checked all the search results to reduce hits with partial conserved-domains and other false positives, using BioEdit (v7.0.5) [65]. Sequences were accepted from BLAST results as long as they shared at least 35 % identity, and had an expected threshold lower than 1e-40 with the conserved domain in the 2OGDs family. Meanwhile, the gene prediction and annotation analysis was considered for the GAox homologs acquisition. Then, 854 GAox homologous were obtained from 41 species; these covered all the main lineages of plants including unicellular green algae, bryophytes, pteridophytes, gymnosperms and angiosperms. Additionally, two ACOs (1-aminocyclopropane-1-carboxylate) of rice were included in phylogenetic analyses considering both ACOs and GA oxidases belong to the 2OGD superfamily.

### Multiple sequence alignment and phylogenetic analysis

A protein multiple sequence alignment (MSA) was generated using MUSCLE (Multiple Sequence Comparison by Log-Expectation) software [66] with the default settings. Experimentally characterized GAox genes of *Arabidopsis thaliana* and *Oryza sativa* were used as criteria to detect potential problems such as frame shift mutations. Protein-coding DNA sequences (CDS) were aligned based on the protein alignments in the DAMBE with the default parameters, then converted the CDS alignments into PAML format for further analyses [67, 68].

RAxML 7.2.6 (Randomized Axelerated Maximum Likelihood) [69] was used to constructed maximum-likelihood (ML) phylogenetic trees under the JTT amino acid substitution model which was derived from ProtTest 2.4 [70], with an estimated gamma distribution parameter, and optimized starting with a BIONJ tree. Statistical support for the nodes on the maximum-likelihood tree was evaluated by bootstrap analysis with 500 iterations.

### Gene structure, motif identification and homology modeling analysis

To study the evolution of the GAox gene structure, the DNA sequences corresponding to each predicted gene and the gene structure of the GAox gene family were all retrieved from http://www.phytozome.net.

To obtain the characteristic motif of each GAox subfamily, the following steps were taken: 1) the web-based multiple expectation maximization for motif elicitation (MEME) analysis (http://meme-suite.org/, [71]) was employed with the following parameters: maximum number of motifs = 15, and optimum motif widths constrained to between 6 and 30 residues. 2) The NCBI-CDD (Conserved Domain Database) (http://www.ncbi.nlm.nih.gov/Structure/cdd/cdd.shtml) and the SMART (Simple Modular Architecture Research Tool, http://smart.embl-heidelberg.de/) databases were used for identification and function explanation of those putative motifs [72]. 3) Multiple sequence alignment of the GAox genes was achieved using MUSCLE software, to find the characteristic motifs of every GAox subfamily. 4) To examine the relationship between the characteristic motif of every GAox subfamily and the putative functional region, the 3D structures of eight OsGAox genes, i.e., *OsGA2ox7*, *OsGA2ox5*, *OsGA20ox4*, *OsGA3ox2*, *OsGA20ox5*, *OsGA20ox6*, *OsGA20ox7* and *OsGA20ox8*, were predicted following the homology modeling using the Swiss-Model (http://swissmodel.expasy.org/) with the default parameters, and the higher reliability PDBs were selected as templates according to the global quality estimation (GMQE) and qualitative model energy analysis (QMEAN) scores [73–75].

Three-dimensional model images with the characteristic motifs and the putative positive selection and functional divergence sites were manipulated and rendered in PyMOL (http://pymol.sourceforge.net/) [76]. All the sequence logos were generated using the online Weblogo platform (http://weblogo.berkeley.edu/).

### Testing positive selection and functional divergence

We used the programs PAML 4.8, FunDi and GroupSim to detect sites involved in the functional divergence of GAox sequences and the adaptive evolution of GAox subfamilies. Sites identified as being functionally divergent or positively selective by all or two of these three programs were highlighted in the 3D protein structure of GA oxidases, which could be tested with direct experimental techniques in the future.

In order to reduce computation time and the potential impact of synonymous site saturation due to sequence divergence [77], the positive selection test was conducted on the site and branch-site models with 46 less divergent GAox homologs from six grass species of monocots. Two ACOs of rice were added in phylogenetic analysis of GAox genes. We performed our analysis using alignments inferred by phylogeny-aware multiple sequence alignment program Probabilistic Alignment Kit (PRANK) [78, 79], which has been reported to outperform other aligners in simulations [80]. In addition, by GUIDANCE filtering [81], the PRANK alignment has a higher average GUIDANCE score of 0.70, while the MAFFT, MUSCLE and CLUSTALW alignments is 0.65, 0.63 and 0.66 respectively, though the GUIDANCE cannot tell which alignment is better.

Selective pressure was tested based on the phylogeny of GAox genes by comparing the nonsynonymous/synonymous substitution ratios (*ω* = *d*_*N*_/*d*_*S*_) with *ω* = 1, < 1, and > 1, which indicate neutral evolution, purifying selection, and positive selection, respectively. To test the functional divergence of different GAox subfamilies, the codeml program in the PAML 4.8 package was used to investigate possible selection acting on different gene subfamilies [82]. We selected F3X4 as codon frequency model, as it accounts for the most important feature of the mutation process, the unequal base frequencies, and the correction for transition/transversion bias is obtained by ML estimation of the kappa parameter [82–85]. The equilibrium frequencies of codons and nucleotides among subfamilies are measured by the DAMBE. Considering the fact that positive selection often operates episodically on a few amino acid sites in a small number of lineages of a phylogenetic tree [86, 87], the modified branch-site model A was run for each gene in each lineage across the phylogenetic tree of the monocots. The LRT (likelihood ratio test) is a general method for testing assumptions (model parameters) through comparison of two competing hypotheses. We used branch-site model A to construct branch-site test 2, which is also called the branch-site test of positive selection. The null and alternative hypotheses are as follows: Null hypothesis (branch site model A, with *ω*2 = 1 fixed): model = 2; NSsites = 2; fix_omega = 1; omega = 1. Alternative hypothesis (branch site model A, with *ω*2 estimated): model = 2; NSsites = 2; fix_omega = 0; omega = 1.5 (or any value > 1). Because of very high sequence divergence, and the likelihood of saturation of synonymous changes, we treated the branch-site test as an exploratory analysis. Additional analyses at the codon level (sites models) and amino acid level (described below) were employed to more formally assess the hypothesis that GAoxs have been subject to functional divergence following the major gene duplication events. Therefore, we analyzed separately each of eight subfamilies (less divergent) under the sites models, only with GAox homologs of angiosperms. Unlike branch-site model, site-specific models allowing *ω* to vary among sites [55, 88, 89] were used to determine whether particular amino-acid residues within GAox families have been subject to positive selection. We use an LRT comparing M0 (one-ratio) with M3 (discrete) (κ = 3) to test for variable selective pressure among sites, and two LRTs to test for sites evolving by positive selection, comparing: M1a (Nearly Neutral) against M2a (Positive Selection), and M7 (beta) (κ = 10) against M8 (beta & ω) (κ = 10). The PP (posterior probability) for the sites under positive selection was calculated by the Bayes empirical Bayes (BEB) method [90].

**Fig. 1:**
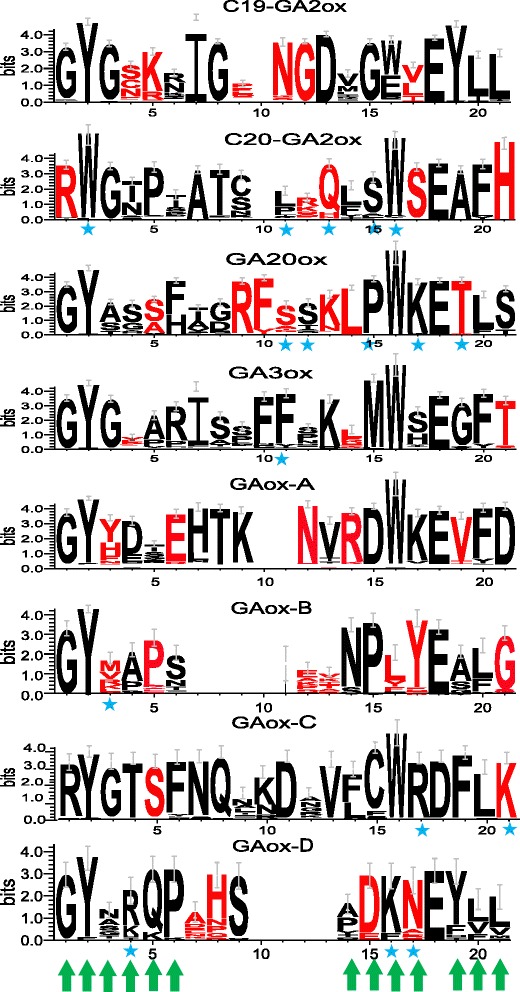
Specific conserved domains of the eight GA oxidase subfamilies and sites predicted to be functionally divergent by at least two of the three programs (PAML 4.8, FunDi, and GroupSim). The overall height of each stack indicates the sequence conservation at that position, whereas the height of symbols within each stack reflects the relative frequency of the corresponding amino acid. Green arrows mean that the sites in these columns are under functional divergence detected by GroupSim with PP ≥ 99 %; the sites highlighted in red are under functional divergence detected by FunDi with PP ≥ 99 %; the sites highlighted with blue star are positive selected sites detected by PAML 4.8 with PP ≥ 95 %

**Fig. 2:**
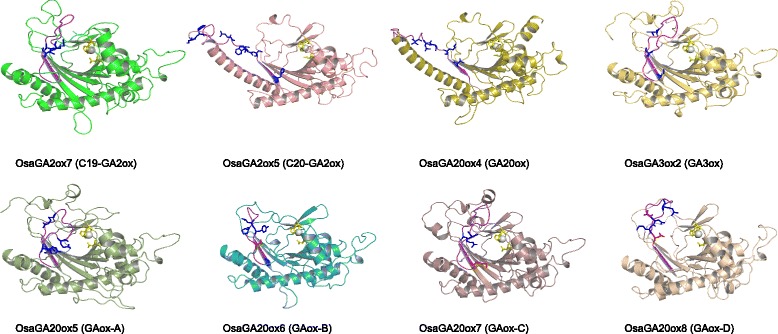
The structure of the eight OsaGAoxs. The characteristic conserved domain of each gene is highlighted in *purple*, amino acid residues that in the characteristic conserved domains identified by two and three programs are highlighted in *blue* and *red*, respectively. Residues in that bind the active-site Fe (*yellow ball*) and those that interact with the 5-carboxylate of 2-oxoglutarate are highlighted in *yellow*

Default options were used in the GroupSim and FunDi using QmmRAxML [91]. In order to evaluate the predictions made by FunDi, we analyzed all the comparisons of eight GAox subfamilies, except for the C_19_-GAox/C_20_-GA2ox and C_20_-GA2ox/GAox-C, due to their large datasets (gene homologs and the alignments) and computation cost (Additional file 1: Table S3). The sites of each subfamily motif were highlighted with red if they occurred in more than five comparisons (Fig. 1). Generally, sites with a functional divergence score above 0.5 were considered to be functionally divergent in order to identify the maximum number of possible candidate sites for consideration [60, 62]. In addition, we analyzed the subfamily characteristic motifs, and mapped those sites detected by two or three programs (PAML 4.8, FunDi and GroupSim) on the 3D protein structures (Figs. 1 and 2). We found that these sites are those positively selected sites detected by the branch-site model of PAML 4.8 with PP ≥ 95 % and those under functional divergence predicted by FunDi and GroupSim with PP ≥ 99 %.

## Results

### Identification and distribution of GAox genes in plants

**Fig. 3:**
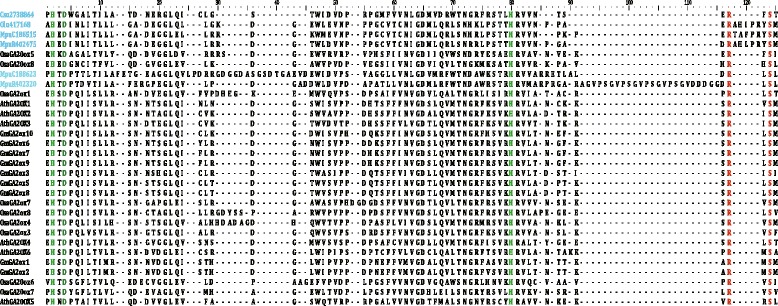
Active site core of 2-oxoglutarate-dependent dioxygenases. Amino acid residues that bind the active-site Fe and those that interact with the 5-carboxylate of 2-oxoglutarate are highlighted in *green* and *red*, respectively. Six GAox homologs from algae are highlighted in *blue*

Our BLAST search results revealed 854 sequences of GAox homologs from the 41 species with whole genome sequencing data; these represent the main lineages of green plants (Additional file 2: Table S2). In algae, 6 putative GAox homologs were found based on the catalytic core amino acid sequences of 2OGDs in the carboxyl terminus (Fig. 3). As shown in Additional file 2: Table S2, the GAox genes varied across the different species and plant taxonomic groups, and there exist different distribution patterns for GAox subfamilies, especially for GAox-A, GAox-B, GAox-C and GAox-D. For instance, all 37 land plants have GAox gene homologs from the subfamilies C_19_-GA2ox, C_20_-GA2ox, GA20ox and GA3ox, while other subfamily genes are not present in one or more taxa, especially in some basal lineages of the land plants. In *Physcomitrella patens* genome, putative GAox homologs were only found in C_19_-GA2ox, GA3ox and GAox-C. There were no homologs of C_20_-GA2ox and GAox-B in a pteridophytes *Selaginella moellendorffii*. Homologs of C_20_-GA2ox and GA20ox were not found in a gymnosperm *Picea abies*. Of angiosperms, monocotyledons have all eight GAox subfamily homologs, while basal angiosperm *Amborella trichopoda* and some eudicotyledons have no GAox-D homologs in their genome. In addition, not only the distribution pattern but the gene sequence and structure were also varied between GAox genes of algae and land plants (Additional file 3: Data S1 and Additional file 4: Figure S1).

### Phylogenetic analysis of plant GAox genes

**Fig. 4:**
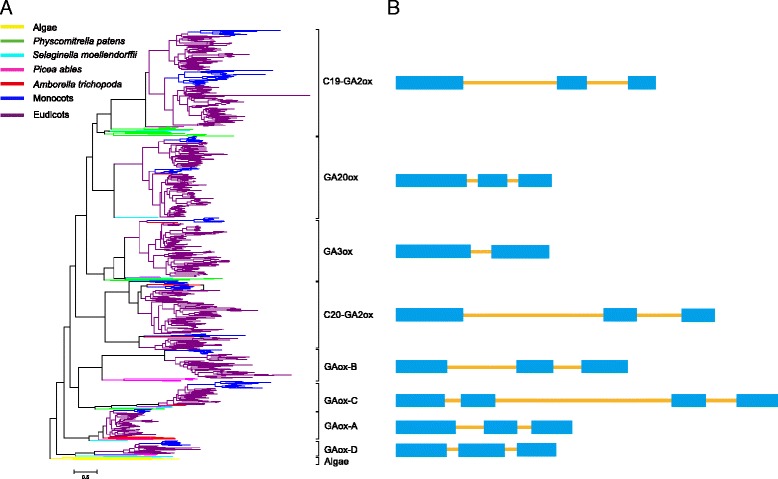
Phylogenetic tree and gene structure of the GA oxidase in plants. **a** The maximum likelihood tree of 41 species’ GA oxidase genes constructed on 854 deduced full-length peptide amino acid sequences and rooted with homologous sequences of algae. **b** The sketch map of GA oxidases structure: Exons and introns are indicated by *blue boxes* and *yellow lines*, respectively. For more details of the GA oxidase structures, see Additional file 4

According to the phylogenetic analysis of 854 GAox homologs, the maximum-likelihood (ML) tree showed that all GAox homologs from green algae were clustered into a single clade (Alga clade), while those from land plants formed another main clade (Embryophyta clade), which was further divided into eight subclades with high statistical support, corresponding to the subfamilies C_19_-GA2ox, C_20_-GA2ox, GA20ox, GA3ox, GAox-A, GAox-B, GAox-C and GAox-D, respectively (Fig. 4a). Out of those, the latter four subfamilies corresponding to *OsGA20ox5*, *OsGA20ox6*, *OsGA20ox7* and *OsGA20ox8* reported in Han and Zhu’s study (2011) were characterized for the first time. Each of these new subfamilies (i.e., GAox-A, GAox-B, GAox-C and GAox-D) formed a monophyletic group, respectively (Fig. 4a), and they are phylogenetically distant from the GA20ox subfamily. GAox homologs from *Physcomitrella patens*, *Selaginella moellendorffii*, *Picea abies* and *Amborella trichopoda* resided in the basal lineages of the tree, which is consistent with previous studies on the phylogenetic position of these species. In addition, for angiosperm species, subfamilies C_19_-GA2ox, C_20_-GA2ox, and GA20ox exhibited a distinct binary branching pattern in the phylogenetic tree, which suggests that large scale genome duplications may have played a critical role in the evolution of these gene subfamilies.

### Characteristic motifs of GAox subfamilies

When we used MEME to identify and examine the conserved motifs of GAoxs, we found a total of 15 conserved motifs (Table 1). Among these 15 motifs, 13 were shared by most GAoxs, and motifs 1 and 2 represent the catalytic core of 2OGDs. Motifs 7, 9, 11 and 12, were found in different GAoxs. For example, motif 7 was absent from all the GA20ox and GA3ox genes, whereas motifs 9 and 11 were not observed in any of the GA2ox genes. Interestingly, motifs 7, 9 and 12 were all localized in N-terminals of the amino acid sequences. The alignment of N-terminal amino acid sequences of all GAox gene homologs revealed eight types of gene sequence (Fig. 1 and Additional file 3: Data S1), which correspond to the C_19_-GA2ox, C_20_-GA2ox, GA20ox, GA3ox, GAox-A, GAox-B, GAox-C and GAox-D subfamilies, respectively. Figure 1 shows that there are 16 to 21 amino acids in the characteristic motifs specific to the eight subfamilies of the GAox family, and the motifs of subfamilies GAox-A, GAox-B, GAox-C and GAox-D have more variable amino acid sites compared with the others. The subfamilies C_19_-GA2ox, C_20_-GA2ox, GA20ox and GA3ox have been studied previously, while GAox-A, GAox-B, GAox-C and GAox-D are proposed for the first time in this study; they include the newly named genes *OsGA2ox5*, *OsGA20ox5*, *OsGA20ox6*, *OsGA20ox7* and *OsGA20ox8* in rice, respectively [23].

**Table 1:** Motif distribution in GA oxidases

Motif number	Length (aa)	Conserved sequence
motif1	30	PGAFVVNVGDTLQALSNGRFKSVLHRVVVN
motif2	18	HTDPTILTILHQDQVGGL
motif3	30	ARLVVKACEEWGFFQVVNHGVPAELISRAE
motif4	19	FEESDSILRLNHYPPCPEP
motif5	19	RLYRDFTWSEYLEFTQKHY
motif6	19	RLSMAYFLGPPLDKVISPL
motif7	30	GPPDPFGYGSKRIGPNGDVGWLEYLLLNTN
motif8	19	VVEEYCEAMKKLALKLLEL
motif9	14	RFSSKLPWKETLSF
motif10	30	DRFFALPLSQKQKAQRSPGEVCGYASAFIG
motif11	14	RADMNTLDAFSNWL
motif12	30	LSNGSYRWGTPTATSLRQLSWSEAFHIPLT
motif13	19	SVKDYFRKTWGNDFEQFGK
motif14	19	SEAYREHPLHLKHIIPLDF
motif15	10	LGDNRLGPFE

Comparative gene structure analysis of 854 GAox gene sequences revealed that gene homologs of every GAox subfamily often have similar exon-intron structures, but the gene structure is more variable in subfamilies C_19_-GA2ox and C_20_-GA2ox, which had two kinds of these structures (Fig. 4b and Additional file 4: Figure S1). Most GAox gene subfamilies have three exons, but this is not the case in subfamilies GAox-C and GA3ox. The GA3ox genes have two exons, and the GAox-C genes generally have four exons. However, in monocots of subfamily GAox-C, three exons were also found in some GAox homologs.

### Positive selection and functional divergence among GAox subfamilies

**Fig. 5:**
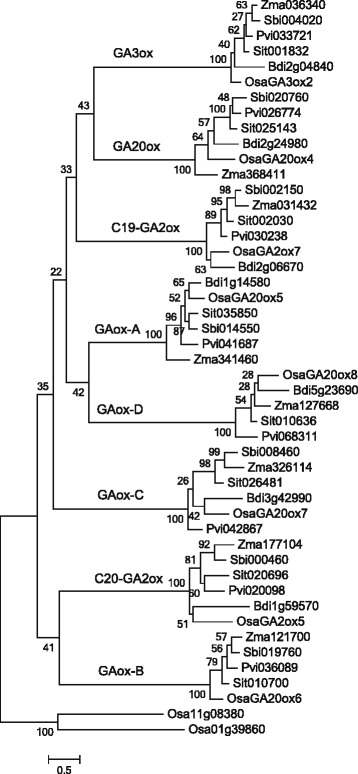
ML tree of the GA oxidases used to test positive selection. Numbers on the tree represent bootstrap values

The phylogenetic trees were constructed based on the sequences of 46 monocots and two ACOs in order to investigate selection pressure among eight GAox subfamilies and selected amino acid sites in characteristic motifs. The LRTs for M2 vs. M1 (2Δ*L* = 0, *p* > 0.05) suggested that the positive selection model (M2) was not significantly better than the nearly neutral model (M1). But models M3 and M8 fit the data significantly better than the null models M0 and M7 (for M3 vs. M0, 2Δ*L* = 1946.50, *p* < 0.001; for M8 vs. M7, 2Δ*L* = 244.99, *p* < 0.001), they identify several sites with an *ω* value significantly greater than 1. At the PP >95 % level, 13 amino acid sites were identified under positive selection by M8 (Table 2). As an exploratory analysis, the branch-site test suggested that all eight GAox subfamilies were under positive selection (*p* < 0.05) (Fig. 5). Bayes Empirical Bayes (BEB) analysis showed that at the PP > 95 %, branch-site model A identified 2/13/29/8/1/12/2/29 sites as being potentially subjected to positive selection on the C_19_-GA2ox/C_20_-GA2ox/GA20ox/GA3ox/GAox-A/GAox-B/GAox-C/GAox-D branches, respecitvely.

**Table 2:** Summary statistics for detecting selection using branch-site and site models of PAML4.8

Model	*p*	*L*	Estimate of parameters	df	2Δ*L*	Positively Selected Sites^a^
Site-specific models						
M0: one-ratio	1	−33,700	*ω*: 0.160			None
M1a: nearly neutral	2	−33,148	*p*_0_ = 0.727, *p*_1_ = 0.273 *ω*_0_ = 0.125, *ω*_1_ = 1.00			Not allowed
M2a: positive selection	4	−33,148	*p*_0_ = 0.727, *p*_1_ = 0.182, *p*_2_ = 0.090; *ω*_0_ = 0.125, *ω*_1_ = 1.000, *ω*_2_ = 1.000	2	0	
M3: discrete	5	−32,727	*p*_0_ = 0.212, *p*_1_ = 0.594, *p*_2_ = 0.194; *ω*_0_ = 0.038, *ω*_1_ = 0.180, *ω*_2_ = 1.934	4	1946.50^***^	21,24,25,27,28 ~ 43,44,45,46,47,48,49,51 ~ 57,60,103,105,106,134,136,137,138,243,279,325,361,362,373,492,493,494,548,549,550 ~ 554,556,557,558,560,561,562,563,566,567
M7: *β*	2	−32,705	*p* = 0.642, *q* = 1.628			Not allowed
M8: *β & ω*	4	−32,583	*p*_0_ = 0.858, *p* = 0.971, *q* = 4.418, *p*_1_ = 0.142, *ω* = 4.858	2	244.99^***^	21,24,25,29,32,35,37,39,40,42,46,49,53,54,56,57,103,134,362,493,550,551,563.
Branch-site model						
model A (C19-GA2ox)	4	−33,132	*p*_0_ = 0.509, *p*_1_ = 0.189, *p*_2_ = 0.302, *ω*_2_ = 8.762	1	6.26^*^	393,520
model A (C20-GA2ox)	4	−33,129	*p*_0_ = 0.446, *p*_1_ = 0.167, *p*_2_ = 0.386, *ω*_2_ = 47.691	1	15.83^***^	150,184,217,232,239,287,289,294,299,383,519,531,535
model A (GA20ox)	4	−33,110	*p*_0_ = 0.442, *p*_1_ = 0.163, *p*_2_ = 0.395, *ω*_2_ = 999.000	1	39.77^***^	65,71,81,114,148,149,159,161,172,190,214,264,291,304,330,348,377,381,384,385,417,437,447,471,477,507,515,519,524
model A (GA3ox)	4	−33,132	*p*_0_ = 0.466, *p*_1_ = 0.175, *p*_2_ = 0.358, *ω*_2_ = 7.881	1	9.51^**^	156,158,163,232,288,339,505,525
model A (GAox-A)	4	−33,135	*p*_0_ = 0.556, *p*_1_ = 0.205, *p*_2_ = 0.238, *ω*_2_ = 13.489	1	10.41^**^	340
model A (GAox-B)	4	−33,133	*p*_0_ = 0.464, *p*_1_ = 0.174, *p*_2_ = 0.363, *ω*_2_ = 39.904	1	14.41^***^	197,231,343,352,353,354,416,444,469,471,525,530
model A (GAox-C)	4	−33,136	*p*_0_ = 0.465, *p*_1_ = 0.174, *p*_2_ = 0.362, *ω*_2_ = 5.019	1	3.97^*^	342,439
model A (GAox-D)	4	−33,124	*p*_0_ = 0.424, *p*_1_ = 0.160, *p*_2_ = 0.417, *ω*_2_ = 21.542	1	17.55^***^	84,160,161,213,231,233,259,263,299,304,310,335,339,341,347,352,364,366,423,427,453,457,470,475,485,511,517,525,545

*p* is the number of free parameters in the *ω* distribution

*significant at *p* value < 0.05 level, **significant at *p* value < 0.01 level, ***significant at *p* value < 0.001 level

^a^positively selected sites identified under site and branch-site tests with PP ≥ 95 %

The separate analyses of the eight subfamilies under the site models showed that the selective pressure could vary among amino acid sites in eight subfamilies by comparison of the M0 and M3 for all GAox subfamilies. The M3 detected no sites under positive selection in most of subfamilies, but both M1a against M2a, and M7 against M8 suggested the presence of sites under positive selection in each subfamily, except for the GAox-D. No sites were found in the GAox-D, possibly due to its small sample size (Additional file 1: Table S3).

The programs FunDi and GroupSim identified a number of sites with significant functional divergence scores for all subfamilies [60–62]. FunDi method predicted a wider range of site divergences, and the fractions of sites with posterior probability of functional divergence above 0.5 ranges from 0.798 to 0.987 across GAox subfamilies (Table 3). The GroupSim method detected 159 sites in all GA oxidases (Table 4).

**Table 3:** Fraction of sites predicted to be functionally divergent by FunDi program

Data sets	FunDi (PP ≥ 95 %)
3ox Vs 20ox7	0.895522388
20ox5 Vs 20ox6	0.987641607
20ox Vs 20ox6	0.96789424
3ox Vs 20ox6	0.970978441
C19 Vs 20ox6	0.962149886
20ox6 Vs 20ox7	0.954333644
C20 Vs 20ox6	0.980555556
C19 Vs 3ox	0.941255908
C19 Vs 20ox7	0.914821124
C19 Vs 20ox	0.928341385
C19 Vs 20ox8	0.960144928
3ox Vs 20ox8	0.926456543
20ox8 Vs 20ox7	0.876175549
20ox Vs 20ox8	0.899563319
20ox8 Vs 20ox6	0.981299213
20ox5 Vs 20ox8	0.798290598
C20 Vs 20ox8	0.960727969
C20 Vs 3ox	0.94165536
C20 Vs 20ox	0.931743421
20ox Vs 20ox7	0.864295125
3ox Vs 20ox	0.897810219
C20 Vs 20ox5	0.952252252
C19 Vs 20ox5	0.930674264
3ox Vs 20ox5	0.926660915
20ox5 Vs 20ox7	0.808544304
20ox Vs 20ox5	0.879332478

**Table 4:** Number of sites predicted to be functionally divergent by GroupSim program

Posterior probability	In characteristic motifs	In GAox family
0.99 ≤ PP	13	127
0.5 ≤ PP < 0.99	1	32

In order to analyze the characteristics and functional divergence sites of each subfamily motif, we modeled the 3D structures of eight GA oxidases of *Oryza sativa* based on the higher reliability templates (Additional file 5: Table S1). According to the 3D protein structures, we found: 1) all the characteristic motifs locate in the crevice of GA oxidases; 2) each of the characteristic motifs consists of a loop and β sheet; 3) all the motifs detected have several positively selected or functional divergence sites by all or two programs (PAML 4.8, FunDi and GroupSim), and most of these sites are polar amino acids (Fig. 2). Those features suggested that the characteristic motif is an important component of GA oxidases and plays a significant role in the GA biosynthesis.

## Discussion

### Origin and evolution of GAox family

The present study showed that GAox genes, critical genes in regulating GA biosynthesis especially for the later steps of GA biosynthetic pathways, were ubiquitous in plants. Unlike the four previously well-studied subfamilies (i.e., C_19_-GA2ox, C_20_-GA2ox, GA20ox and GA3ox), there were fewer homologs in the subfamilies GAox-A, GAox-B, GAox-C and GAox-D ranging from 30 to 63 (Additional file 2: Table S2). Homologs of subfamilies GAox-B and GAox-D genes were not found in the basal angiosperm *Amborella trichopoda*, but were present in the gymnosperm *Picea abies*. Our phylogenetic analysis of the GAox genes showed that all six GAox homologs identified from green algae fell into a single clade, while those from land plants were clustered together and then divided into eight subclades (Fig. 4a). Previous studies found no bioactive GAs have been detected and chemically identified in mosses, nor could external GAs induce GA-specific developmental processes in these plants [27, 92, 93]. However, Anterola and Shanle (2008) found 15 putative GA oxidase homologs in *Physcomitrella patens*. This means that there are GA oxidases, but no bioactive GAs in moss species, which could be due to incompletion of GA synthetic pathway. In this study, we found 18 putative GAox genes in *Physcomitrella patens*, belonging to three different subfamilies. These results indicate that diversification of GAox genes occurred before the divergence of land plants, then diversified and expanded further in the course of land plant evolution. Considering GAox genes in the main plant lineages, it is reasonable to believe that a rapid gene expansion occurred in the GAox family after the divergence of land plants from the common ancestor of green plants; this may have been related to specialized metabolisms and adaptations to terrestrial environments.

According to our phylogenetic analysis, gene structure and the characteristic motifs of GAox genes, eight subfamilies were well defined under the GAox family of land plants, namely C_19_-GA2ox, C_20_-GA2ox, GA20ox, GA3ox, GAox-A, GAox-B, GAox-C and GAox-D. Of these subfamilies, C_19_-GA2ox, C_20_-GA2ox, GA20ox and GA3ox have been thoroughly studied previously, while new subfamilies GAox-A, GAox-B, GAox-C and GAox-D have not. According to our phylogenetic analysis, these four subfamilies resided in basal clades in the tree. All this information suggests that these subfamilies of genes were present in the common ancestral species of extant land plants, and these genes may have some unknown functions in land plants. Thus, the catalytic activity and biological function of these genes needs to be elucidated.

**Fig. 6:**
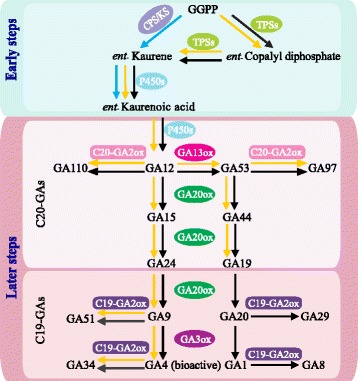
Comparison of the major gibberellin (GA) biosynthesis pathways in *Physcomitrella patens* (bryophyte), *Selaginella moellendorffii* (pteridophyte) and seed plants. The biosynthesis pathways of bryophyte, pteridophyte and seed plants are highlighted in *blue*, *orange* and *black arrows*, respectively. Abbreviations: CPS, ent-copalyl diphosphate synthase; GGDP, geranylgeranyl diphosphate; KS, ent-kaurene synthase; KO, ent-kaurene oxidase; KAO, entkaurenoic; P450s, cytochrome P450 monooxygenases; TPSs, terpene synthases

Although the structures and functional sites were predicted in our study, the function of GAox-A/B/C/D genes has not been examined yet. Therefore, it is unknown the location of these genes in GA biosynthesis pathway. Combined with the findings of previous studies [11, 40, 92, 94], a proposed model of GAs biosynthesis pathway is presented in Fig. 6. Obviously, only very early steps of this pathway were present in *Physcomitrella patens*. Biochemically, P450s (cytochrome P450 monooxygenases) provide direct substrates or precursors of substrates for C_20_-GA2ox, so the presence of C_20_-GA2ox may be a prerequisite for the presence of the GAox family. As the GAox-A/B/C/D are basal clades of phylogenetic tree (Fig. 4), they may appear subsequently during the evolution of land plants. However, it is currently difficult to give a clear explanation for the evolutionary history of GAox genes due to lack of information of causal relationship between active GAs and GAox-A/B/C/D.

Despite having similar catalytic cores of 2OGDs (Fig. 3), the GAox homologs of green algae exhibited some differences to those of the nonvascular plant *Physcomitrella patens* and vascular plants. They were grouped into one clade in the phylogenetic tree, and not classified into any of GAox subfamilies. In addition, there are no characteristic motifs of eight GAox subfamilies. These findings suggest that putative algal GAox genes have little diversification of sequences and functions.

### Diversification of gene structure

Previous studies have shown that gene structural diversity is a possible mechanism for the evolution of multigene families [95–97]. The GAox family provides an example of exon-intron diversity in the evolution of gene family, although the protein sequences of its catalytic domain are well conserved. Comparing the phylogenetic tree with the exon-intron structure (Fig. 4 and Additional file 4: Figure S1), it is apparent that the most closely related members within subfamilies shared similar gene structure in terms of intron number and exon length, suggesting that the diversification of subfamilies could be under a strong selection and indicative of their functional conservativeness. Most of the GAox subfamilies have three exons, also suggesting that the diversification of the GAox family is under a strong selection. However, two recent diverged GAox subfamilies, GA3ox and GAox-C possess two and four exons, respectively; this supports the hypothesis that duplications of ancestral mosaic genes have been followed by more recent gains and losses of introns [98]. In view of the numbers and locations of introns, GAox genes may have experienced different evolutionary histories since the monocot-dicot split. Unlike the gene structure of dicots, that of monocots is usually simple, without or with one/two shorter introns, suggestive of the fact that these genes probably arose from retrotransposon-based random insertions. This might indicate different evolutionary patterns for these two kinds of plants. Most homologs in the three GAox subfamilies (i.e. C_19_-GA2ox, C_20_-GA2ox and GAox-C subfamily) with relatively longer introns may have arisen from unequal crossing-over [23].

To elucidate the motif diversification of GAox subfamilies, putative motifs were predicted using MEME and alignments (Table 1). As expected, most of the closely related members had common motif compositions, and the length and location of GAox gene domains were highly conserved, suggesting similar functions among members of the same GAox subfamily and the same selection acting on them. All proteins of the eight subfamilies have very similar C-terminal structures of their catalytic domains (Fig. 3 and Additional file 3: Data S1), the function of most putative motifs remains unknown as they do not have homologs in the Pfam and SMART databases. Most of the motifs were shared by the majority of GAoxs, but the short amino acid stretches in the N-terminal may characterize each subfamily. Combined with the previous studies of GAox [35–39, 45, 46], we conclude that the alignment of amino acid sequence from 691 to 757 sites can be used to identify a putative protein of the GAox family (Fig. 1 and Additional file 3: Data S1). In addition, these motifs can be used to distinguish subfamily C_20_-GA2ox from C_19_-GA2ox [20]. According to the results of 3D modeling analysis, all characteristic motifs were located in the crevice of the protein surface (Fig. 2). In addition, the positive selection tests suggest that most of these motifs contain several sights that could have evolved in response to positive selection pressure. To date, no functional examination has been conducted on these conserved motifs, so our results provide evidence that these specific motifs may have some active sites and play critical roles in GA biosynthesis and catabolism.

Based on our findings, the similar gene structures and conserved motifs in each GAox subfamily provide an insight into the evolutionary history of the GAox family and its classification. In addition, the variation in gene structure and the difference in motif compositions among different subfamilies indicate that GAox subfamilies are functionally diversified. Furthermore, the eight conserved motifs are the diagnostic characters for the identification of GAox subfamilies, and they must be important for catalytic functions in the GA biosynthetic pathway.

### Function divergence and adaptation of the GAox family

Gene duplication is considered to be a major mechanism for the generation of evolutionary novelty and adaptation [50, 51]. In plants, gene duplication followed by functional divergence is particularly important for the diversification of biochemical metabolites [99–102]. Substitutions can change the function of duplicated genes, and may be due to either a relaxation of purifying selection or the action of positive selection [56, 58, 59, 103]. To investigate evidence for positive selection in the GAox subfamilies, we analyzed the eight branches by site models and branch-site models (Fig. 5 and Additional file 1: Table S3). Further analyses on each of the subfamilies under the site models (M0 vs M3; M1a vs M2a; M7 vs M8) detected several positively selected sites for all the subfamilies except for the GAox-D, suggesting that the selective pressure varies among lineages and amino acid sites in GA oxidases. The biochemical context of substitutions that was under positive selection is consistent with a scenario involving the adaptive evolution of GAox genes. Under branch-site models, some selected sites (PP > 95 %) were found to be scattered throughout the primary sequences (data not shown), and they were folded in the large cavity of three-dimensional structures of the analyzed protein. Among these sites, one to eight positively selected sites (PP > 95 %) were located in or near the characteristic motifs, in all branches except C_19_-GA2ox and GAox-A. The mutations at these sites among the different subfamilies suggested their importance for the enzymatic activity in GA biosynthesis and catabolism. Further studies using site-directed mutagenesis are needed to determine whether these selected sites confer an ability on GAox genes to discriminate between different substrate types.

It should be noted that our branch-site model analyses might suffer from uncertainty because of limitations of the codon models, such as changes in equilibrium nucleotide and codon frequencies among clades with deep divergence (Additional file 6: Table S5) [85, 104, 105]. Therefore, we further performed FunDi and GroupSim to investigate the divergence of the distribution of site-rates. Because these methods are more appropriate for the high sequence divergences, they could provide an important cross-check of the codon-based results and confirmed that the GA oxidases underwent functional divergence. Despite this, the signal for positive selection contributing to the divergence of GA oxidases should be taken with caution considering that the codon and amino acid models can sometimes be negatively affected by model misspecification and alignments quality [83, 86, 106–110]. Taken together, our study provides new insights into the functional divergence of the GAox family and implicates the potential roles of positive selection in the evolution of GAox genes, which deserves further investigations.

## Conclusions

This study provides the first large-scale evolutionary analysis of GAox genes in plants, using an extensive whole-genome dataset of 41 species representing green algae, bryophytes, pteridophytes and seed plants. Our study not only provides information about the classification of GAox genes, but also facilitates the further functional characterization and analysis of GA oxidases. Our results indicate: 1) Gibberellin oxidase (GAox) originated very early—before the divergence of bryophytes and the vascular plants, and diversified into eight subfamilies in the course of land plant evolution. Of these subfamilies, GAox-A, GAox-B, GAox-C and GAox-D are proposed for the first time; 2) the diversification of GAox genes could be attributed to functional divergence and such divergence is most likely to facilitate the completion of the GA synthesis pathway for plants to adapt to terrestrial environments; 3) each subfamily of GAox genes has its characteristic motif and a signature of positive selection have been detected in most subfamilies.

### Availability of supporting data

Additional file 3-Data S1 and Additional file 7-Data S2. Protein sequences used in the present analysis.

Additional file 8-Table S4. GenBank accessions numbers for sequences used in this study.

## Additional file**s**

Additional file 1: Table S3. MLEs of parameters and sites inferred to be under positive selection for the eight GAox subfamilies data set. (XLSX 15 kb) Additional file 2: Table S2. GA oxidases in the species analyzed in this study. (XLSX 19 kb) Additional file 3: Data S1. Alignment of putative GA oxidase sequences. (FASTA 1761 kb) Additional file 4: Figure S1. The GA oxidase structure. (PDF 1521 kb) Additional file 5: Table S1. List of templates used in this study. (XLSX 10 kb) Additional file 6: Table S5. A. Frequencies of nucleotides; B. Frequencies of codons; C. Nucleotide frequencies by codon sites. (RAR 24 kb) Additional file 7: Data S2. The aligned (protein-coding) DNA sequences (CDS). (RAR 238 kb) Additional file 8: Table S4. The accession numbers of the protein sequences used in the present analysis. (XLSX 34 kb)

## Abbreviations

ACOs: 1-aminocyclopropane-1-carboxylate
CPS: Ent-copalyl diphosphate synthase
GA: Gibberellin
GAox: GA oxidase
GGDP: Geranylgeranyl diphosphate
KAO: Entkaurenoic
KO: Ent-kaurene oxidase
KS: Ent-kaurene synthase
LRT: Likelihood ratio test
P450s: Cytochrome P450 monooxygenases
TPSs: Terpene synthases
2OGD: 2-oxoglutarate-dependent dioxygenase
