# Supplementary material for: Divergence and adaptive evolution of the gibberellin oxidase genes in plants
Source: BMC Evol Biol. 2015 Sep 29;15:207. doi: 10.1186/s12862-015-0490-2 (PMC4587577; doi:10.1186/s12862-015-0490-2)
Supplement: Additional file 4: Figure S1. — The GA oxidase structure. (PDF 1521 kb) [file 12862_2015_490_MOESM4_ESM.pdf]

Figure S1

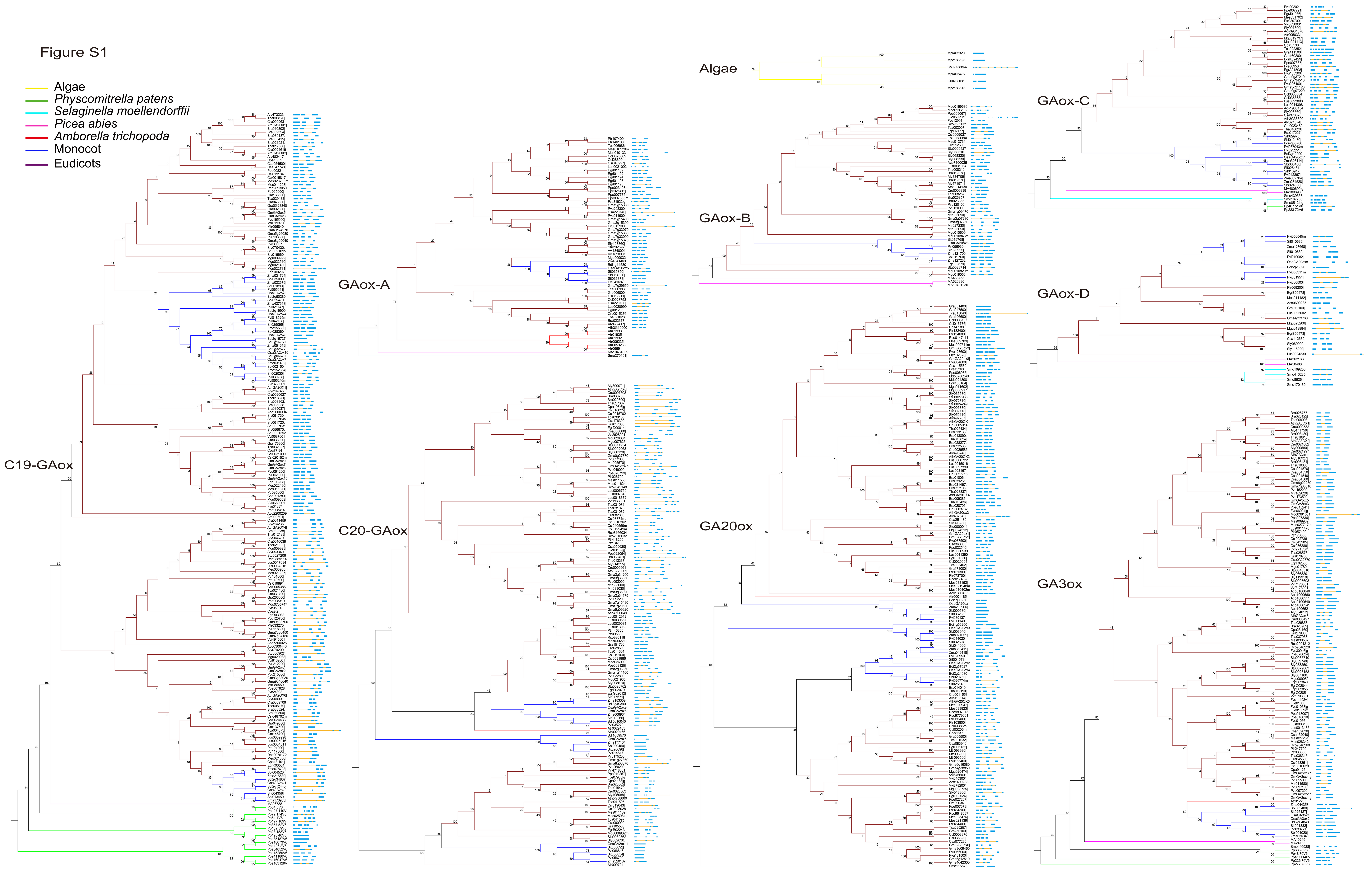
Algae
Physcomitrella patens
Selaginella moellendorffii
Picea abies
Amborella trichopoda
Monocot
Eudicots
C19-GAox
GAox-A
C20-GAox
Algae
GAox-B
GA20ox
GAox-C
GAox-D
GA3ox
